# Upregulation of miR-664a-3p Ameliorates Calcific Aortic Valve Disease by Inhibiting the BMP2 Signaling Pathway

**DOI:** 10.1155/2022/2074356

**Published:** 2022-10-07

**Authors:** Yun Jiang, Wei Ji, Jiaqi Zhu, Zihao Shen, Jianle Chen

**Affiliations:** ^1^Department of Burn and Plastic Surgery, Affiliated Hospital of Nantong University, Medical School of Nantong University, Nantong, Jiangsu 226000, China; ^2^Department of Thoracic Surgery, Rudong People's Hospital, Nantong, Jiangsu 226400, China; ^3^Department of Cardiothoracic Surgery, Affiliated Hospital of Nantong University, Medical School of Nantong University, Nantong, Jiangsu 226000, China

## Abstract

The development of calcific aortic valve disease (CAVD) is a complex process of ectopic calcification involving various factors that lead to aortic valve stenosis, hemodynamic changes, and, in severe cases, even sudden death. Currently, aortic valve replacement is the only effective method. The osteogenic differentiation of aortic valve interstitial cells (AVICs) is one of the key factors of valve calcification. Emerging evidence suggests that bone morphogenetic protein 2 (BMP2) can induce the proosteogenic activation of AVICs. However, the regulatory mechanism underlying this activation in AVICs is unclear. In the present study, we elucidated through high-throughput RNA sequencing and RT-qPCR that miR-664a-3p was evidently downregulated in the calcific aortic valve. We also proved that miR-664a-3p was involved in regulating osteogenic differentiation in AVICs. Target prediction analysis and dual-luciferase reporter gene assay confirmed that miR-664a-3p is preferentially bound to BMP2. Furthermore, the effect of the miR-664a-3p/BMP2 axis on osteogenic differentiation in AVICs was examined using the gain- and loss-of-function approach. Finally, we constructed a mouse CAVD model and verified the effect of the miR-664a-3p/BMP2 axis on the aortic valve calcification leaflets *in vivo*. In conclusion, miR-664a-3p regulates osteogenic differentiation in AVICs through negative regulation of BMP2, highlighting that miR-664a-3p may be a potential therapeutic target for CAVD.

## 1. Introduction

Calcified aortic valve disease (CAVD) is a high-risk disease in older people and is closely related to morbidity and mortality in patients with cardiovascular disease. It is also a major risk factor for cardiovascular disease complications, such as myocardial infarction [1–3]. So far, there are still no effective clinical interventions to reverse CAVD or halt its progression [4–6]. Valve replacement is the only effective clinical option [7]. Identification of the pathological mechanisms of CAVD can help its treatment. Aortic valve calcification is an active process involving complex changes, such as endothelial injury, lipid infiltration, chronic inflammation, matrix remodeling, cell differentiation, calcium salt deposition, and neovascularization [8]. Moreover, the active regulation of CAVD formation involves the synergistic action of multiple cells, including resident valvular endothelial cells, valvular interstitial cells, bone marrow-derived cells, and circulating inflammatory and immune cells [9]. Among them, valvular interstitial cells (VICs) play an important role in maintaining normal valve structure and function [10]. The activation of VICs in the normal dormant state is a major mechanism of the pathological process of aortic valve calcification. VICs can change to the osteoblast phenotype and accumulate calcium, phosphorus, and other inorganic salt ions, causing the cells to calcify [11]. The process of VIC transition may involve multiple signal transduction pathways [3, 12]. Therefore, the strategy of preventing VIC transformation by inhibiting osteoblast differentiation may lead to new therapeutic interventions to prevent and even reverse CAVD progression.

Previous studies have shown BMP2 as an important proosteogenic factor involved in vascular and aortic valve calcification [13–15]. Inorganic phosphate osteogenic induction medium promotes VIC osteogenic differentiation via the BMP2 signaling pathway [16]. A previous study also claimed that concurrent upregulation of BMP2 and TGF-*β*1 is responsible for biglycan-induced proosteogenic reprogramming in human aortic VICs (AVICs) [17]. Therefore, the osteogenic effect of BMP2 on AVICs may play an important role in aortic valve calcification and CAVD progression. Studying the regulatory pathway of BMP2-induced osteogenic differentiation of AVICs will advance our understanding of the molecular mechanism of CAVD occurrence and development.

In the search for effective therapeutics to treat CAVD, microRNA is an exciting candidate, as its expression can be manipulated using microRNA mimics or inhibitors [18]. MicroRNAs are a class of noncoding RNA with a size of 18–22 nt [19, 20]. They not mainly regulate the degradation of target mRNA but also regulate the translational inhibition depending on complementarity between the miRNA and mRNA [21]. MicroRNAs can regulate various physiological and pathological processes, such as cell proliferation, development, differentiation, and apoptosis; in fact, microRNA dysfunction often leads to impaired cell function [22–25]. Numerous studies have shown that the level of microRNAs changes dramatically during the osteogenic differentiation process in CAVD. For example, miR-138 suppresses the osteoblastic differentiation of VICs in degenerative CAVD [26]. miR-214 was found to inhibit aortic valve calcification in stretch-induced CAVD [27]. Moreover, miR-34a improved aortic valve calcification by regulating the Notch1-runt-related transcription factor 2 (Runx2) signaling pathway [28]. These studies indicate that microRNAs play a dual role in the process of aortic valve calcification. Previous studies have shown that microRNA regulates osteogenic differentiation by regulating BMP2 expression. For instance, BMP2 downregulated miR-30b and miR-30c to increase RUNX2 expression in human coronary artery smooth muscle cells and promote mineralization [19]. However, whether microRNA regulates the osteogenic differentiation of AVICs via the BMP2 pathway remains unclear.

In the present study, we first compared the expression of microRNAs between the calcified aortic valve leaflets of CAVD patients and normal tissues through RNA sequencing. Subsequently, the results were verified by gain- and loss-of-function experiments to examine whether miR-664 participates in regulating AVIC osteogenic differentiation by influencing BMP2 expression. Finally, we constructed an animal model of CAVD and verified the effect of the miR-664a-3p/BMP2 axis on the calcification of aortic valve leaflets *in vivo*. Our findings show that miR-664a-3p participates in regulating AVIC osteogenic differentiation by negatively regulating BMP2 expression, highlighting that miR-664a-3p may be a potential therapeutic target for CAVD.

## 2. Materials and Methods

### 2.1. Clinical Sample Collection

A total of 16 pairs of tissue samples were obtained from Jiangsu Province Hospital, China, including calcified aortic valve leaflets (CAVs), obtained via aortic valve replacement procedures and normal noncalcified aortic valves without thickening or nodules isolated via heart transplantation procedures. Samples with diseases such as rheumatic aortic valvulopathy, congenital valve disease, infective endocarditis, and autoimmune disease were excluded. All protocols using patient samples were approved by the Ethical Committee of Jiangsu Province Hospital. Written informed consent was obtained from the patients before surgery. All studies involving humans were conducted in accordance with the Helsinki Declaration as well as relevant guidelines and regulations.

### 2.2. RNA Extraction and Sequencing

MicroRNA was extracted and purified from human CAVs or normal aortic valve tissue by using the miRNeasy Mini Kit (217004; Qiagen, Germany). A small RNA Sample Prep Kit (RS200-0012; Illumina, Germany) was used to construct the microRNA library. MicroRNA expression was processed for 50 bp single-end reads using the miRDeep2 analysis pipeline. The cDNA was amplified by reverse transcription using primers that were complementary to the 3′ junction, and the microRNA library was obtained by 15 cycles of PCR amplification using the Illumina HiSeq 2000 high-throughput sequencing technology. The detailed operation was performed by Shanghai Kangcheng Biological Company. MicroRNA sequences were assigned names consistent with miRBase 20. R packages, DESeq2 and EdgeR, were utilized to normalize counts and calculate differential expression of microRNAs [29, 30].

### 2.3. Isolation of Human Aortic Valve Interstitial Cells and Cell Culture

Human AVICs were isolated from noncalcified heart valves using collagenase I, as described previously [28]. In brief, valve leaflets were digested in a medium containing 1.0 mg/mL collagenase type I for 30 min at 37°C. Valve endothelial cells were removed, and 1.0 mg/mL collagenase I fresh medium was added for 4-6 h at 37°C. After vortexing and repeated aspiration to break up the tissue mass, the cells were precipitated by centrifugation at 1000 rpm for 10 min. The temperature during centrifugation was 4°. Finally, the precipitated cells were resuspended and cultured in Dulbecco's Modified Eagle Medium (DMEM, Thermo Fisher, USA) containing 10% fetal bovine serum (Thermo Fisher) and 100 U/mL penicillin-streptomycin (Sigma, USA). Next, the cells were incubated in an osteogenic induction medium for 14 days to stimulate osteogenic differentiation, as previously described [31]. Complete DMEM was supplemented with 0.25 mmol/L L-ascorbic acid, 10 mmol/L *β*-glycerophosphate, and 10 nmol/L dexamethasone (Sigma).

### 2.4. Lentivirus Infection

BMP2 shRNA (F: 5′- AATTCAAAAAGTTCGAGTTGCGGCTGCTCAGCTCGAGCTGAGCAGCCGCAACTCGAAC-3′, R: 5′- CCGGGTTCGAGTTGCGGCTGCTCAGCTCGAGCTGAGCAGCCGCAACTCGAACTTTTTG-3′), scramble (F: 5′-AATTCAAAAAGCGCGATAGCGCTAATAATTTCTCGAGAAATTATTAGCGCTATCGCGC-3′, R: 5′-CCGGGCGCGATAGCGCTAATAATTTCTCGAGAAATTATTAGCGCTATCGCGCTTTTTG-3′), miR-664a-3p mimic/inhibitor (mimic-F: 5′- AUAAGUAAAUAGGGGUCGGAUGU-3′, mimic-R: 5′- AUCCGACCCCUAUUUACUUAUUU-3′, inhibitor: 5′- AUAAGUAAAUAGGGGUCGGAUGU-3′), and the corresponding control (mimics-NC-F: 5′-GAGAUGUUCAAUCGGGUAUUU-3′, mimics-NC-R: 5′- AUACCCGAUUGAACAUCUCUU-3′, inhibitor-NC: 5′- GAAUUACAUGCACCACUCAAU-3′) primers were all synthesized by Shanghai Gene Pharma. shRNA was inserted into the pLKO-TRC plasmid (Sigma-Aldrich). Human and mouse BMP2/Bmp2 coding sequences (human: F: 5′--GGGAGAAGGAGGAGGCAAAG --3′, R: 5′--GAAGCAGCAACGCTAGAAGACA--3′; mouse: F: 5′--GACATCCGCTCCACAAACGA--3′, R: 5′--CATCACTGAAGTCCACATACAAAGG--3′) were inserted into the overexpressed pcDNA3.1 plasmid vector (Invitrogen). All lentiviral solutions were obtained from Gene Pharma.

VICs (1 × 10^5^ cells/mL) were seeded in a 6-well plate and cultured in an osteogenic medium. After overnight incubation at 37°C for wall attachment, 200 *μ*L of 1 × 10^8^ transduction units (TU)/mL lentiviral solution was added. The medium was replaced after 20 h of infection. After 48 h of infection, 3 ng/*μ*L puromycin (Solarbio) was added to screen for successful infection of cells.

### 2.5. Construction of Calcific Aortic Valve Disease Animal Model

A CAVD animal model was established as described previously [32]. Male ApoE^−/−^ (C57BL/6 background) mice aged 6–8 weeks were purchased from Nanjing University Animal Center, China, and housed under specific pathogen-free conditions and 12 h dark-light cycle in individually ventilated cages at 22°C, with free access to food and water. The mice were fed a 0.2% high cholesterol diet for 24 weeks to develop aortic valve calcification [33]. Next, the mice were randomly divided into four groups of 12 mice per group, namely, control, miR-664a-3p mimic, Bmp2 overexpression (OE), and miR − 664a − 3p mimic + Bmp2 OE. The lentivirus of miR-664a-3p and Bmp2 OE vectors was obtained from GenePharma (Shanghai, China). The lentiviruses were injected into the mice via the tail vein twice a week for another 10 weeks. Animal care and euthanasia were carried out with the approval of the Institutional Animal Care and Use Committee of Nanjing Medical University.

### 2.6. Western Blotting Analysis

Western blotting analysis was performed based on previous reports [34]. Protein was extracted from VICs and aortic valve tissues using RIPA lysis buffer with protease inhibitors (Thermo Fisher). Next, 20 *μ*g of the protein solution was fractionated by 10% SDS-PAGE and then transferred to a polyvinylidene difluoride membrane (Millipore). After blocking with 5% nonfat milk, the membrane was incubated at 4°C overnight with primary antibodies, including anti-BMP2, anti-RUNX2, anti-MST2, anti-OSTERIX, and anti-GAPDH (as an internal reference). Subsequently, the membrane was washed with Tris-buffered saline-Tween 20 (TBST) solution and incubated with goat antirabbit HRP-conjugated secondary antibody for 2 h at room temperature. All antibodies were purchased from Abcam and used in accordance with the manufacturer's instructions. Protein bands were visualized using an enhanced chemiluminescence kit (Vazyme) and quantified using the ImageJ software.

### 2.7. Reverse Transcription-Quantitative Polymerase Chain Reaction (RT-qPCR)

Total RNA was isolated from VICs or aortic valve tissues using a miRNeasy Mini Kit (Qiagen) and then reverse-transcribed to cDNA using the PrimeScript RT Master Mix (Takara, Japan) with oligo (dT) or random primer. After that, RT-qPCR was performed using the PowerUp SYBR Green Master Mix (Thermo Scientific, USA) on an Applied Biosystems 7500 Fast Real-Time detection system (Applied Biosystems). U6 or GAPDH gene was used as the internal control for measuring relative gene expression. All experiments were independently repeated three times [35, 36]. The primer sequences for miR-664a-3p and U6 are listed in Table S1.

### 2.8. Alizarin Red Staining

VICs were washed by PBS and fixed with 4% PFA for 30 min. The cells were subsequently stained with 2% Alizarin Red S solution (Sigma) at room temperature for 30 min. Red staining indicates the formation of calcified nodules. Next, the cells were washed by PBS and observed under a light microscope (Olympus). Quantitative analysis was performed using the ImageJ software.

### 2.9. Alkaline Phosphatase (ALP) Activity Assay

VICs were washed twice with PBS. Proteins were then extracted with 1% Triton X-100 and centrifuged at 10,000 rpm for 10 min. ALP activity was measured using the 5-bromo-4-chloro-3-indolyl phosphate (BCIP)/nitro blue tetrazolium (NBT) alkaline phosphodiesterase chromogenic Kit (C3206; Beyotime) according to the manufacturer's protocol. The stained samples were observed under a light microscope (Olympus). Quantitative analysis was performed using the ImageJ software.

### 2.10. Hematoxylin-Eosin (HE) Staining

Mouse and human aortic valves were removed and fixed with 4% paraformaldehyde for 24 h. The tissues were then paraffin-embedded and cut into sections of 5 *μ*m thickness. Subsequently, the sections were dewaxed and stained with HE (C0105S; Beyotime) according to the manufacturer's protocol. Finally, the stained sections were observed under a light microscope (Olympus).

### 2.11. RNA Fluorescent In Situ Hybridization (RNA-FISH)

A Cy3-labeled anti-digoxin miR-664a-3p probe (5′-ATAAGTATAGGGGTCGGATGT-3′) was obtained from GenePhama. VICs cultured in osteogenic differentiation medium were considered as “case,” and those cultured in the normal medium were considered as “control.” The VICs were inoculated at a density of 1 × 10^4^ cells/well in 48-well plates (the wells were pretreated with appropriately sized coverslips). The cells were then mixed and incubated overnight at 37°C in a 5% CO_2_ incubator. The next day, the medium was removed, and the cells were washed twice with PBS. After that, the PBS was removed, and 100 *μ*L of 4% paraformaldehyde was added to each well. The cells were then fixed for 15 min at room temperature. RNA-FISH kit (cell sliver; GenePhama) was used for the subsequent experiments, according to the manufacturer's instructions. Finally, the cell nuclei were stained with 4′-6-diamidino-2-phenylindole (DAPI), and images were captured using a Zeiss LSM 700 confocal microscope (Carl Zeiss).

### 2.12. Luciferase Reporter Assay

BMP2 wild-type 3′-UTR containing putative miR-664a-3p binding sites was inserted into the pGL3 control luciferase reporter vector (Promega, USA). To assess binding specificity, the sequence interacting with miR-664a-3p was mutated by using the Q5® Site-Directed Mutagenesis Kit Protocol (New England Biolabs), and the mutated BMP2 (BMP2-MUT) 3′-UTR was also inserted into the pGL3 plasmid. VICs were cultured in 24-well plates and transfected with Lipofectamine 3000 (Thermo Scientific). Next, 1 *μ*g of luciferase reporter plasmid was added to each well, followed by treatment with 0.2 *μ*g of pRL-TK *Renilla* luciferase plasmid (internal control), 100 pmol/well of either miR-664a-3p mimic, miR-664a-3p inhibitor, or the corresponding control. Luciferase activity was measured at 48 h posttransfection using a dual-luciferase reporter system (Promega). The ratio of firefly to *Renilla* luciferase activity was determined to eliminate variations in transfection efficiency [32].

### 2.13. Statistical Analysis

All experiments were repeated three times. The data were analyzed on SPSS 18.0, and the results are presented as the mean ± SEM. The student's *t*-test was used for the comparison of two value sets. One-way ANOVA or two-way ANOVA followed by Dunnett's T3 post hoc test was conducted to analyze differences between more than two groups. The association between two variables was evaluated by two-tailed Pearson's correlation analysis. GraphPad Prism 7.0 was used for data analysis. *P* < 0.05 indicates a statistically significant result. All measurements were performed in triplicate.

## 3. Results

### 3.1. miR-664a-3p Is Downregulated in Human Aortic Valve Calcification Disease Samples

**Figure 1:**
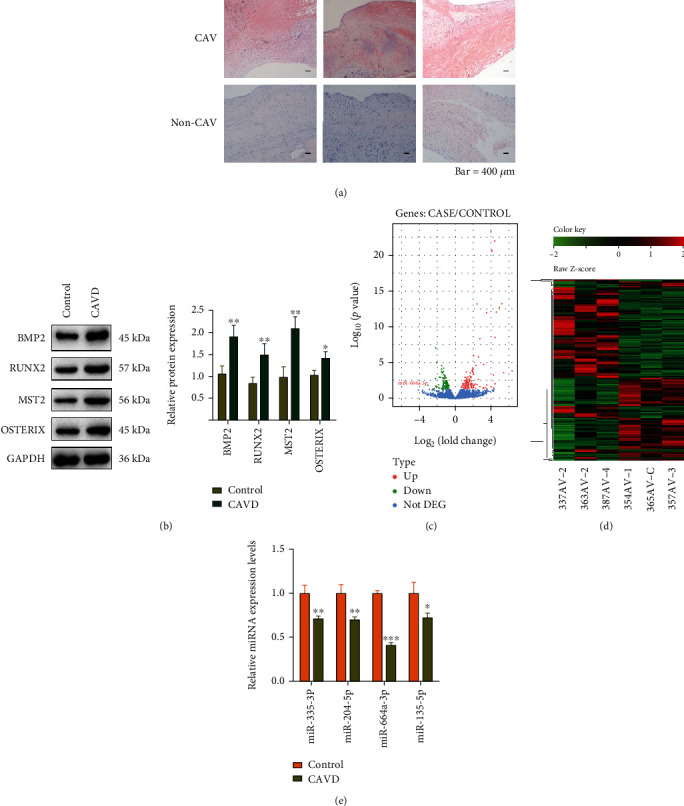
miR-664a-3p is downregulated in human aortic valve calcification disease samples. (a) HE staining of the aortic valve from CAVD and normal clinical samples. Scale bar, 400 *μ*m. (b) Western blotting and quantification of BMP2, RUNX2, MST2, and OSTERIX protein expression in CAVD and normal clinical samples. GAPDH was used for normalization. (c) Volcano plots showing the differential expression of microRNAs in the aortic valve from CAVD clinical samples compared to control samples. Pink dots indicate significantly upregulated microRNAs in the CAVD group. Green dots indicate significantly downregulated microRNAs. Blue dots indicate microRNAs with insignificant differences. (d) Heat map shows the clustering analysis results of the top 20 microRNAs for differential expression in three pairs of samples. The red (higher expression) or green (lower expression) color represents the normalized expression value of the indicated microRNAs. (e) The expression levels of four candidate microRNAs showing the most significant downregulation in CAVD clinical samples were verified by RT-qPCR in five additional pairs of samples. Data are presented as mean ± SEM. ^∗^*P* < 0.05, ^∗∗^*P* < 0.01, and ^∗∗∗^*P* < 0.001.

We first performed HE staining of human CAVs and normal AVs (Figure 1(a)), and the results showed that patients in the CAVD group had calcified tissue lesions, which caused the valve to lose its original uniform dense structure. Furthermore, we performed western blotting analysis to determine the protein expression of osteogenic genes, including BMP2, RUNX2, MST2, and OSTERIX, which are essential for bone formation [37–39]. As shown in Figure 1(b), BMP2, RUNX2, MST2, and OSTERIX were significantly upregulated in CAVD patients. To investigate the role of microRNAs in CAVD, RNA sequencing was performed to identify the difference in microRNA expression between 3 CAVD and normal samples each. The volcano plot showed differential expression of microRNAs, with miR-664a-3p (labeled in the plot; Figure 1(c)) being the most downregulated microRNA in the CAVD group. Clustering analysis of the top 20 differentially-expressed microRNAs was performed, and the results are shown as a heat map, with 4 downregulated and 16 upregulated microRNAs in the CAVD group. qPCR results confirmed that miR-664 was the most downregulated microRNA in CAVD tissues (Figure 1(e)).

### 3.2. miR-664a-3p Inhibits Osteogenic Differentiation and Calcification of Valvular Interstitial Cells

**Figure 2:**
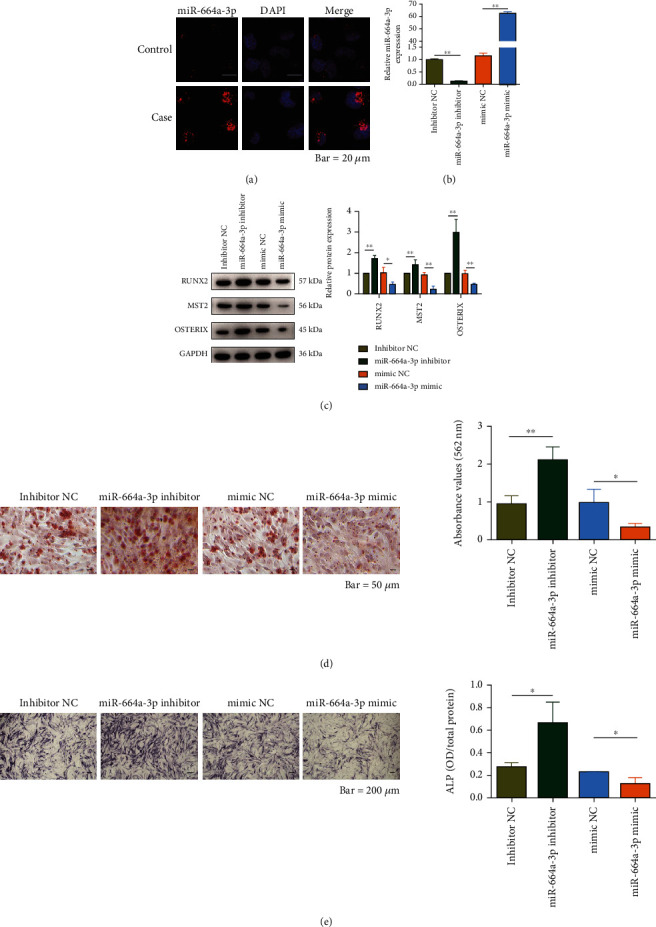
miR-664a-3p inhibits osteogenic differentiation and calcification of valvular interstitial cells. (a) RNA-FISH revealed the localization of miR-664a-3p in VICs. miR-664a-3p is stained in red and the nucleus in blue. Scale bar, 20 *μ*m. (b) RT-qPCR was conducted to detect the miR-664a-3p expression in VICs after overexpression with a mimic or knockdown by an inhibitor. (c) Western blotting analysis of RUNX2, MST2, and OSTERIX expression in VICs after alterations in the level of miR-664a-3p. Quantitative analysis of the protein bands are shown on the right. (d) The formation of calcium nodules after alterations in the level of miR-664a-3p was detected by Alizarin Red staining. Quantitative analysis is shown on the right. Scale bar, 50 *μ*m. (e) ALP activity in VICs after alterations in the level of miR-664a-3p was detected and quantitated by ALP staining assay. Scale bar, 200 *μ*m. ^∗^*P* < 0.05, ^∗∗^*P* < 0.01, and ^∗∗∗^*P* < 0.001.

We assessed changes in the expression of osteogenic differentiation-related indicators to examine whether miR-664a-3p influences the development of CAVD by modulating miR-664a-3p expression in VICs. RNA-FISH analysis results showed that miR-664a-3p was localized in the cytoplasm of VICs. Compared to the control, VICs cultured with osteogenic differentiation medium had a higher level of miR-664a-3p (Figure 2(a)). We used a mimic and an inhibitor to regulate miR-664a-3p expression in VICs and then measured gene expression by RT-qPCR (Figure 2(b)). Western blotting analysis showed a significant negative correlation between the expression of miR-664a-3p and osteogenic differentiation-related proteins, including RUNX2, MST2, and OSTERIX (Figure 2(c)). Alizarin Red staining and ALP activity assay results showed that the downregulation of miR-664a-3p promoted the formation of calcium nodules and increased ALP activity (Figures 2(d) and 2(e)). All these results suggested that miR-664a-3p inhibited the osteogenic differentiation and calcification of VICs.

### 3.3. BMP2 Is a Target Gene of miR-664a-3p

**Figure 3:**
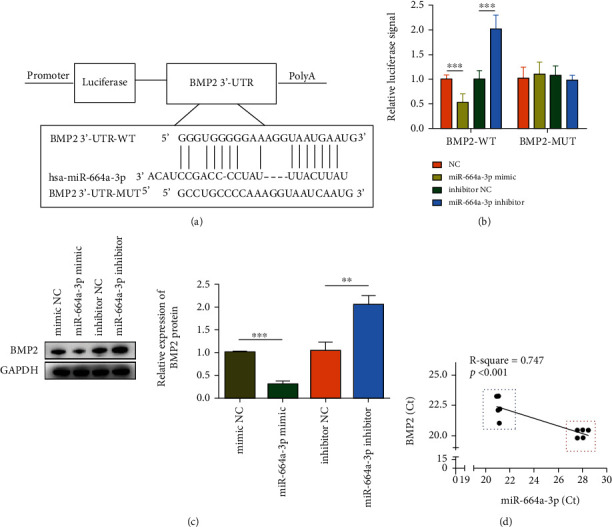
BMP2 is a target gene of miR-664a-3p. (a) Schematic representation of the predicted binding of miR-664a-3p to BMP2. Schematic diagram of the structure of the luciferase reporter system. (b) The relative luciferase activity of VICs coinfected with constructed luciferase reporters (BMP2-WT or BMP2-MUT) and miR-664a-3p mimic or inhibitor or negative control. (c) Western blotting analysis of BMP2 expression in VICs after alterations in the level of miR-664a-3p. Quantitative analysis of the protein bands are shown on the right. (d) Pearson's correlation analysis between BMP2 and miR-664a-3p in human CAVs (five dots in the red box) and human non-CAVs (five dots in the blue box). The horizontal and vertical coordinates indicate the mean Ct values of miR-664a-3p and BMP2 in RT-qPCR, respectively. ^∗^*P* < 0.05, ^∗∗^*P* < 0.01, and ^∗∗∗^*P* < 0.001.

The potential binding site between miR-664a-3p and the 3′-UTR of BMP2 was predicted (Figure 3(a)). A dual-luciferase reporter gene assay was conducted to examine the interaction between miR-664a-3p and BMP2 in VICs. The results showed that luciferase activity was significantly lower in the BMP2-WT group after the expression of miR-664a-3p was enhanced, whereas reducing the level of miR-664a-3p led to an opposite result. However, in the BMP2-MUT group, luciferase activity was not affected by the expression level of miR-664a-3p (Figure 3(b)). RT-qPCR and western blotting analyses revealed that miR-664a-3p negatively regulated the expression of BMP2 (Figures 3(c) and 3(d)). The cycle threshold (Ct) values of miR-664a-3p and BMP2 corresponding to each sample were obtained by RT-qPCR analysis of 5 CAV tissues and normal AV tissues each. Pearson's correlation analysis based on the Ct values revealed a significant negative correlation between the two: miR-664a-3p expression was lower, whereas BMP2 expression was higher in the disease group. As shown in Figure 3(d), the data in red boxes were from CAV tissues, whereas the data in blue boxes were from normal AV tissues. The above results fully confirmed that miR-664a-3p could target BMP2.

### 3.4. BMP2 Promotes the Osteogenic Differentiation and Calcification of Valvular Interstitial Cells

**Figure 4:**
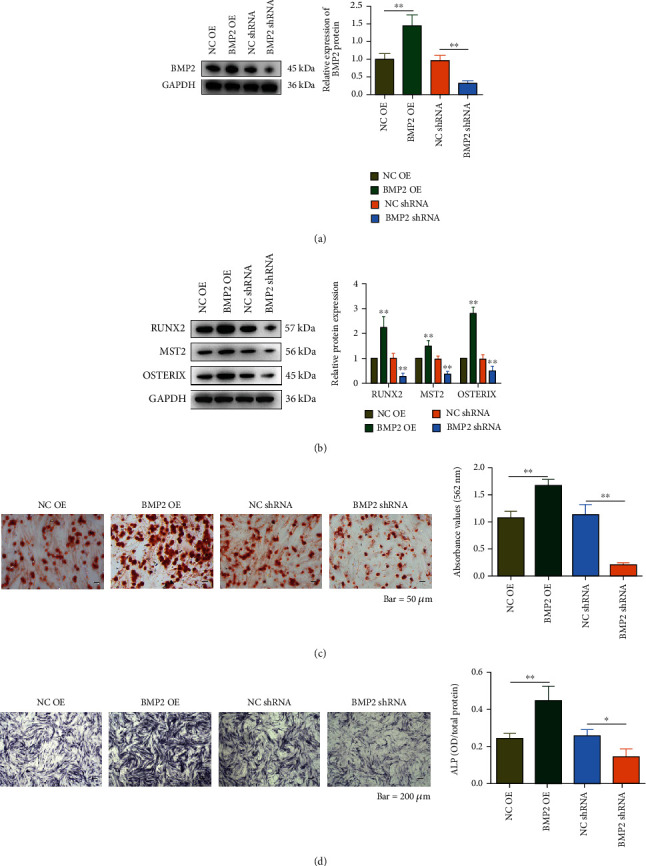
Validation of the effect of BMP2 on osteogenic differentiation in valvular interstitial cells. (a) Detection of BMP2 expression by western blotting after overexpression or knockdown of BMP2 in VICs. Quantitative analyses of the protein bands are shown on the right. (b) RUNX2, MST2, and OSTERIX expression were detected by western blotting analysis after overexpression or knockdown of BMP2 in VICs. Quantitative analyses of the protein bands are shown on the right. (c) Alizarin Red staining was conducted to detect the formation of calcium nodules after alterations in the level of BMP2. The quantitative analysis is shown on the right. Scale bar, 50 *μ*m. (d) ALP activity was detected and quantitated by ALP staining assay after alterations in the level of BMP2. Scale bar, 200 *μ*m. ^∗^*P* < 0.05, ^∗∗^*P* < 0.01, and ^∗∗∗^*P* < 0.001.

Given that BMP2 was overexpressed in CAVs and a target of miR-664a-3p, which was associated with the osteoblast differentiation of VICs, we performed overexpression and knockdown experiments in VICs to investigate whether BMP2 could reprogram VICs toward an osteogenic phenotype. First, western blotting results confirmed that BMP2 expression in VICs was successfully regulated (Figure 4(a)). After the expression of BMP2 was altered, we then examined the expression of other osteogenic differentiation-related proteins (RUNX2, MST2, and OSTERIX), which was positively correlated with BMP2 expression (Figure 4(b)). This result suggested that BMP2 regulated the osteogenic differentiation of VICs by affecting the expression of associated genes. Alizarin Red and ALP staining assays showed that the overexpression of BMP2 enhanced the calcification of VICs, whereas BMP2 knockdown inhibited VIC calcification (Figures 4(c) and 4(d)). Collectively, these results indicated that BMP2 played a positive role in the osteoblast differentiation and calcification of VICs.

### 3.5. miR-664a-3p Inhibits Aortic Valve Calcification by Regulating BMP2 In Vivo

**Figure 5:**
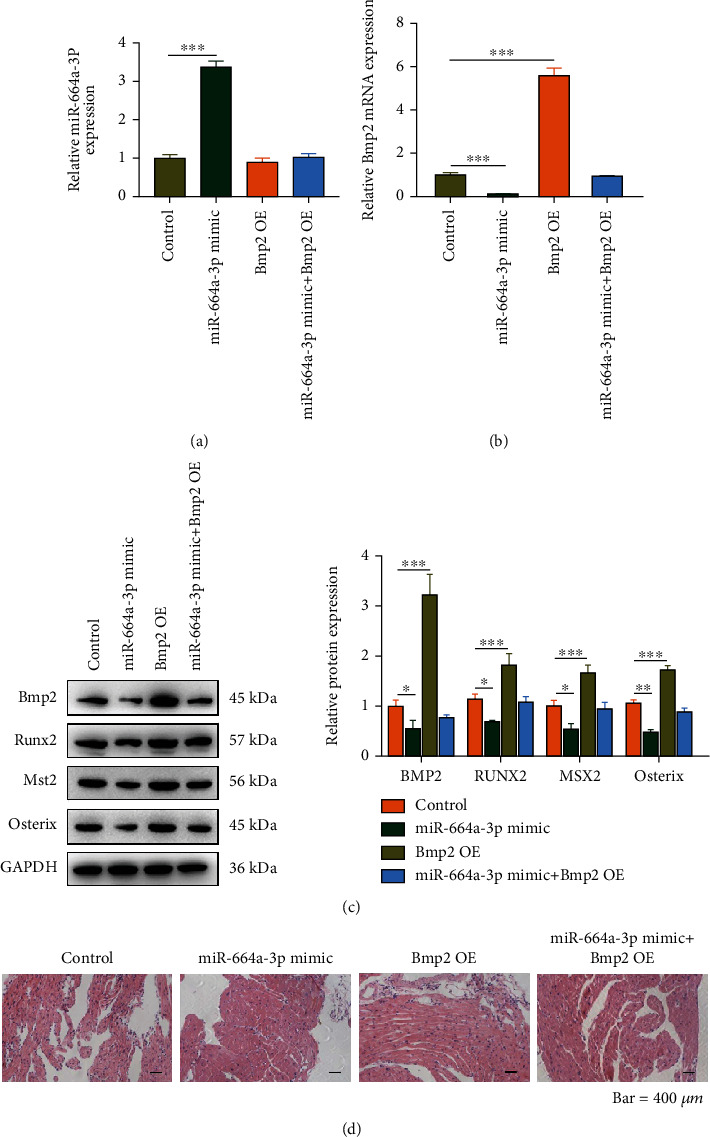
miR-664a-3p regulates aortic valve calcification via Bmp2 in calcific aortic valve disease mice. (a, b) RT-qPCR was performed to measure the miR-664a-3p and Bmp2 expression after upregulation of miR-664a-3p and Bmp2 separately or simultaneously in the aortic valves of mice. (c) Bmp2, Runx2, Mst2, and Osterix expressions were detected by western blotting after the upregulation of miR-664a-3p and Bmp2 levels separately or simultaneously in the aortic valves of mice. Quantitative analyses of the protein bands are shown on the right. (d) HE staining of the aortic valve of mice in different groups. Scale bar, 400 *μ*m. ^∗^*P* < 0.05, ^∗∗^*P* < 0.01, and ^∗∗∗^*P* < 0.001.

**Figure 6:**
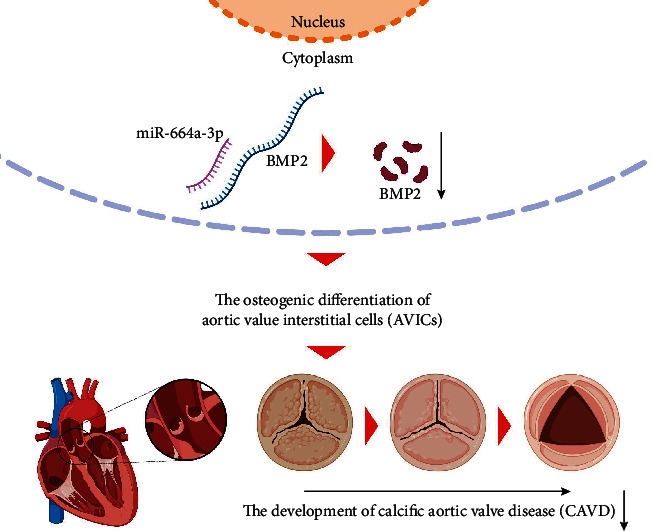
The biological process and mechanism of action of this paper.

To verify the above experimental results, we conducted animal experiments in CAVD -induced mice. We overexpressed miR-664a-3p and Bmp2 separately or simultaneously in CAVD mice by lentiviral transfection. Total RNA was extracted from mouse aortic valve tissues, and RT-qPCR analysis was performed. The results revealed a significant increase in miR-664a-3p and Bmp2 expression after infection with the corresponding virus (Figure 5(a)). In addition, Bmp2 expression was significantly downregulated after infection with the miR-664a-3p overexpression lentivirus (Figure 5(b)). The subsequent western blotting analysis yielded the same result. miR-664a-3p overexpression significantly downregulated the expression of Bmp2, Runx2, Mst2, and Osterix. Moreover, the overexpression of Bmp2 upregulated the expression of Runx2, Mst2, and Osterix; however, this phenomenon was reversed by the upregulation of miR-664a-3p (Figure 5(c)). This observation further suggested that miR-664a-3p also downregulated Bmp2 expression in an *in vivo* setting and that the latter may influence aortic valve leaflets osteogenic differentiation by positively regulating the expression of genes involved in osteogenic differentiation. HE staining of the aortic valve showed a uniform density of valve cells in the miR-664a-3p overexpression group, as well as inflammatory infiltration and new capillary formation in the valve after BMP2 upregulation, whereas the histology of the mice in both overexpression groups was similar to that in the control group (Figure 5(d)). The above experiments showed that miR-664a-3p improved the calcification of aortic valve leaflets by targeting Bmp2 *in vivo*. Finally, the biological process and mechanism of action of this paper are shown in Figure 6.

## 4. Discussion

CAVD is a cardiovascular disease with high morbidity and mortality, especially in the elderly [40, 41]. Studies have shown that calcification plays an important role in the pathogenesis of CAVD, and the osteogenic differentiation of AVICs has been confirmed to be closely related to the pathological process of CAVD [42]. However, there are still no effective pharmacological treatments to prevent or treat this disease. Therefore, the study of the regulatory mechanism of AVIC osteogenic differentiation may contribute to a better understanding of the pathogenesis of CAVD and provide a new perspective for the treatment of the disease.

In the present study, we detected the protein expression of BMP2 in CAVD patient samples and non-CAVD samples. The result showed that the BMP2 protein expression level in CAVD patient samples was significantly higher than that in normal AV samples. This result indicates that BMP2 is indeed involved in the regulation of CAVD and has a posttranscriptional regulation mechanism. RNA sequencing of CAVs and normal tissue revealed many differentially expressed microRNAs, and miR-664a-3p, which was the most downregulated microRNA in calcified valves, was selected for subsequent studies. However, several other microRNAs were also significantly downregulated and could be candidates for follow-up studies. In addition, many other microRNAs were significantly upregulated in CAVD tissues, showing great potential for further study. Although we have not explored these microRNAs, we will continue to study them in-depth in the hope of gaining a more comprehensive understanding of the important role of microRNAs in CAVD development.

Subsequently, we examined the expression of osteogenic differentiation-related proteins after altering miR-664a-3p expression in VICs. Our findings showed that a low expression of miR-664a-3p exacerbated calcification in VICs. miR-664a-3p was found to target and bind to BMP2, thus downregulating BMP2 expression. The relationship between the two was confirmed by dual-luciferase reporter assay and Pearson's correlation analysis. Cardiac valve calcification is the active conversion of VICs to an osteoblast-like cell phenotype, and it involves the regulation of diverse osteogenic factors [43], including the promotion of BMP2 [16]. We again assessed the effect of BMP2 levels on calcification in VICs by altering the expression of BMP2. Not surprisingly, high BMP2 expression exacerbated calcification in VICs, as evidenced by ARS and ALP staining results. In addition, western blotting results showed that BMP2 levels were positively correlated with common osteogenic differentiation-related proteins such as RUNX2, MST2, and OSTERIX. A previous study reported that the formation of the RUNX2-SMAD regulatory complex was obligatory for activating a gene network that drives osteoblast differentiation. As a molecular endpoint, RUNX2 was required to execute and complete TGF-/BMP2 signaling in osteoblasts [44].

Finally, we used ApoE^−/−^ mice to construct a CAVD model by controlling their diet. Next, we upregulated the levels of miR-664a-3p and Bmp2 in the CAVD mice by long-term lentiviral injection. We examined the morphological changes in the aortic valves after incubation for a period of time. Our results showed that a high expression of BMP2 exacerbated aortic valve calcification in mice, whereas the upregulation of miR-664a-3p had the opposite effect. This result confirmed, in an *in vivo* setting, the important role of miR-664a-3p/Bmp2 in the development of aortic valve calcification.

## 5. Conclusion

In conclusion, this study reveals, for the first time, that miR-664a-3p regulates aortic valve calcification by targeting BMP2. The findings of the present study advance mechanistic studies related to the development of CAVD and provide a potential therapeutic target for improving the outcome of CAVD.

## Data Availability

The data used to support the findings of this study are available from the corresponding author upon request.

## Abbreviations

CAVD: Aortic valve calcification disease
AVICs: Aortic valve interstitial cells
BMP2: Bone morphogenetic protein 2
VICs: Valvular interstitial cells
CAVs: Calcified aortic valve leaflets
ALP: Alkaline phosphatase
RT-qPCR: Real-time quantitative polymerase chain reaction
RNA-FISH: RNA fluorescent in situ hybridization
WT: Wild-type
MUT: Mutated.

## Additional Points

*Research Highlights.* (i) miR-664a-3p is evidently downregulated in the calcific aortic valve. (ii) BMP2 can induce proosteogenic activation of AVICs. (iii) miR-664a-3p regulates AVIC osteogenic differentiation by negatively regulating BMP2. (iv) miR-664a-3p may be a potential therapeutic target for CAVD.

## Supplementary Materials

Supplementary Materials Table-S1: primer sequences.
