# Supplementary material for: Upregulation of miR-664a-3p Ameliorates Calcific Aortic Valve Disease by Inhibiting the BMP2 Signaling Pathway
Source: Dis Markers. 2022 Oct 7;2022:2074356. doi: 10.1155/2022/2074356 (PMC9568341; doi:10.1155/2022/2074356)
Supplement: Supplementary Materials — Table-S1: primer sequences. [file 2074356.f1.docx]

| **Table-S1: Primer sequences** | |
| --- | --- |
| **Human** | **Mouse** |
| miR-664a-3p reverse transcription primer:  miR-664a-3p5'--GTCGTATCCAGTGCAGGGTCCGAGGTATTCGCACTGGATACGACTGTAGG--3'  QPCR primer sequences：  miR-664a-3p-F: 5'-- CGCGTATTCATTTATCCCCAG --3'  miR-664a-3p-R: 5'-- AGTGCAGGGTCCGAGGTATT --3'    U6-F: 5'--GGCAGCACATATACTAAAATTG --3'  U6-R: 5'--GGAACGCTTCACGAATTTGCG --3' | miR-664a-3p reverse transcription primer:  miR-664a-3p5'--GTCGTATCCAGTGCAGGGTCCGAGGTATTCGCACTGGATACGACTGTAGG--3'  QPCR primer sequences：  miR-664a-3p-F: 5'--CGCGTATTCATTTATCCCCAG--3'  miR-664a-3p-R: 5'--AGTGCAGGGTCCGAGGTATT--3'    U6-F: 5'--CTCGCTTCGGCAGCACATATACT--3'  U6-R: 5'--ACGCTTCACGAATTTGCGTGTC--3' |
|  |  |
